# Moderate decline in select synaptic markers in the prefrontal cortex (BA9) of patients with Alzheimer’s disease at various cognitive stages

**DOI:** 10.1038/s41598-018-19154-y

**Published:** 2018-01-17

**Authors:** Odile Poirel, Sébastien Mella, Catherine Videau, Lauriane Ramet, Maria Antonietta Davoli, Etienne Herzog, Pavel Katsel, Naguib Mechawar, Vahram Haroutunian, Jacques Epelbaum, Stéphanie Daumas, Salah El Mestikawy

**Affiliations:** 1 Sorbonne Universités, UPMC Univ Paris 06, INSERM, CNRS, Neurosciences Paris Seine - Institut de Biologie Paris Seine (NPS - IBPS), 75005 Paris, France; 2 Université Sorbonne Paris Cité, UMR-S894 Inserm Université Paris Descartes, Centre de Psychiatrie et Neuroscience, 75014 Paris, France; 3 Douglas Hospital Research Center, Department of Psychiatry, McGill University, 6875 boulevard Lasalle Verdun, Quebec, QC Canada; 4 Mount Sinai School of Medicine, Department of Psychiatry, NewYork, NY USA; 5 MECADEV UMR 7179 CNRS, Muséum National d’Histoire Naturelle, 91800 Brunoy, France; 6 Present Address: Universités Bordeaux, CNRS, IINS, UMR 5297, F-33000 Bordeaux, France

## Abstract

Synaptic loss, plaques and neurofibrillary tangles are viewed as hallmarks of Alzheimer’s disease (AD). This study investigated synaptic markers in neocortical Brodmann area 9 (BA9) samples from 171 subjects with and without AD at different levels of cognitive impairment. The expression levels of vesicular glutamate transporters (VGLUT1&2), glutamate uptake site (EAAT2), post-synaptic density protein of 95 kD (PSD95), vesicular GABA/glycine transporter (VIAAT), somatostatin (som), synaptophysin and choline acetyl transferase (ChAT) were evaluated. VGLUT2 and EAAT2 were unaffected by dementia. The VGLUT1, PSD95, VIAAT, som, ChAT and synaptophysin expression levels significantly decreased as dementia progressed. The maximal decrease varied between 12% (synaptophysin) and 42% (som). VGLUT1 was more strongly correlated with dementia than all of the other markers (polyserial correlation = −0.41). Principal component analysis using these markers was unable to differentiate the CDR groups from one another. Therefore, the status of the major synaptic markers in BA9 does not seem to be linked to the cognitive status of AD patients. The findings of this study suggest that the loss of synaptic markers in BA9 is a late event that is only weakly related to AD dementia.

## Introduction

Alzheimer’s disease (AD) is characterized by a progressive and severe loss of cognitive abilities. Accumulation of both amyloid beta-peptide (Aβ) deposits in the form of plaques and neurofibrillary tangles composed of hyperphosphorylated tau protein are morphological hallmarks of AD. The pathogenic roles of Aβ and tau are not clearly understood and are still a matter of controversy^1,2^. In addition, heterogeneous and area-specific neuronal and synaptic loss is described in AD^3–11^. A recent meta-analysis reported that the severity of cognitive impairment often correlates with the extent of the synaptic loss^12^. These observations suggest that synaptic loss is a common marker of multiple types of dementia across brain regions^13,14^. Accordingly, AD must have severe effects on the delicate neurochemical balance in brain areas such as the cerebral cortex. Identifying the vulnerability of various neurotransmitter systems in AD could lead to the identification of novel pharmacotherapeutic strategies that aim to alleviate dementia.

Glutamate is the major excitatory neurotransmitter in the brain that is particularly relevant in the cerebral cortex. The fate of glutamatergic neurotransmission in AD has been the subject of numerous reports. In particular, excessive glutamatergic transmission and its accompanying excitotoxicity is often viewed as a key player in the neuronal loss associated with AD and other neurodegenerative pathologies^15–19^. If excitotoxicity is causal in AD, then glutamatergic transmission must increase at some point in AD. This has been reported in animal models following Aβ application^15,17^. Glutamatergic synaptic activity is tightly controlled by excitatory amino acid transporters (EAATs^18,20^). Among the 5 high-affinity glutamate transporters, the major subtype, EAAT2, is mainly expressed in astrocytes^21,22^. EAAT2 is responsible for 90% of the clearance of glutamate from the extracellular space (for review see ref.^21^). Consistent with the excitotoxicity hypothesis of AD, glutamate uptake and EAAT expression are reported to be significantly decreased in AD^23–25^. However, other studies have indicated a decreased density of cortical vesicular glutamate transporters (VGLUTs) in AD^26^ and a mouse model of amyloidosis^27,28^. VGLUTs are pivotal pre-synaptic markers of glutamatergic neurotransmission. VGLUT1 and VGLUT2, the 2 major subtypes, are expressed in cortical and subcortical glutamatergic neurons^29^, and the expression levels of both have been found to decline in AD^23,26,28,30^. Furthermore, in neocortical Brodmann area 9 (BA9), the loss of VGLUT1 strongly correlates with cognitive decline^30^. These data suggest that the disease progression occurs in two stages. During the initial steps of the pathology, increased glutamatergic transmission leads to excitotoxicity and neuronal degeneration. This is followed by a decrease in glutamatergic transmission in AD patients^31^. However, this view is far from having been clearly established in human patients. Furthermore, it is not clear whether and to what extent the density of synaptic markers can be linked to synaptic loss of function^32–35^. For instance, it was recently reported that a massive loss of VGLUT3 (−80%) has only a limited impact on the function of VGLUT3-postive synapses^36^.

The status of both synaptic and neurotransmission markers in AD, as well as in animal models, has been thoroughly investigated. Cholinergic^37^, as well as somatostatin-positive GABAergic interneurons^38,39^, have been reported to be centrally involved in AD and cognitive decline. The loss of cortical cholinergic innervation is considered one hallmark feature of AD^40–42^ and was tentatively linked to attentional and cognitive decline that are associated with the pathology^43,44^. In animal models, the loss of cholinergic neurons in the basal forebrain was shown to be secondary to intraneuronal Aβ accumulation and glutamate excitotoxicity^15,17,45,46^. For decades, GABAergic transmission was considered to be preserved in AD compared to cholinergic and glutamatergic transmission. However, recent animal studies revealed the importance of the GABA/glutamate balance in the pathogenesis of AD^47–50^.

These findings implied that to prevent or treat the dementia associated with AD, synaptic functions should be restored. Furthermore, these data suggest that synaptic proteins can be used as potential biomarkers of the progression of dementia. It is, therefore, of paramount importance to confirm or invalidate these findings. As highlighted by the meta-analysis of de Wilde and colleagues^12^, most studies supporting these conclusions were performed with relatively small sample sizes (on average, n = 10 controls and n = 10 AD patients). The present study sought to address this issue by conducting a thorough post-mortem assessment of synaptic markers in a large group of well-characterized frozen dry-homogenized samples of AD patients. We measured the density of glutamatergic (VGLUT1, VGLUT2, PSD95, and EAAT2), GABAergic (VIAAT and somatostatin) and cholinergic (choline acetyl transferase; ChAT) markers, as well as α-tubulin and synaptophysin, in BA9 samples from 171 individuals stratified according to the clinical dementia rating scale (CDR). Consistent with previous reports, we found that, with the exception of VGLUT2 and EAAT2, all synaptic markers decreased during the late stages of dementia (CDR5). VGLUT1 was the only marker that was significantly diminished in subjects with CDR scores of 3. Furthermore, as has been previously established, VGLUT1 appeared to be the best biomarker of dementia^30^. This study confirmed that a decline in the density of synaptic markers is associated with dementia^3^. However, taken separately, or in linear combination using principal component analysis (PCA), the 7 markers for which a difference between groups was detected (VGLUT1, VIAAT, somatostatin, ChAT, α-tubulin, synaptophysin and PSD95) cannot be used as CDR markers in AD. The present study suggests that synaptic loss in BA9 may be involved in the progression of dementia but in a subtler way than previously thought.

## Results

### Western blot optimization

**Figure 1:**
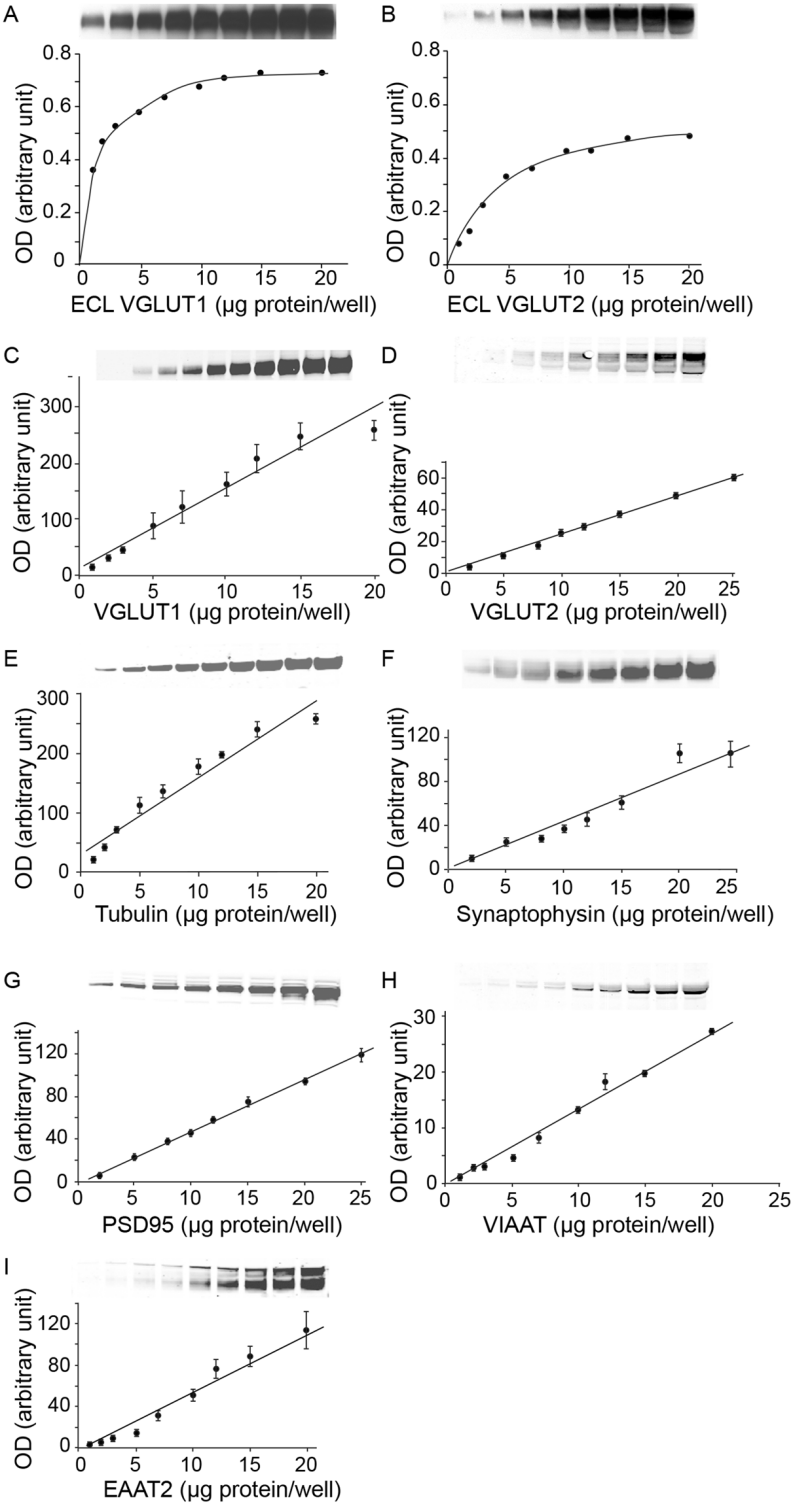
Comparison and linearity of western blot assays coupled to either ECL or infrared detection. Various amounts of BA9 cortical extracts (1–20 µg protein) were separated on SDS-PAGE gel. VGLUT1 (**A**,**C**), VGLUT2 (**B**,**D**), α-tubulin (**E**), synaptophysin (**F**), PSD95 (**G**), VIAAT (**H**) and EAAT2 (**I**) were detected by western blot coupled to ECL (**A**,**B**) or to infrared detection (**C**–**I**).

In this study, 7 biomarkers were detected by western blot. To optimize the western blot detection of the various biomarkers, we compared ECL to IR revelation (Fig. 1). Various amounts of total BA9 extracts (1–20 µg protein) were loaded on SDS-PAGE gels and blotted for VGLUT1 or VGLUT2 detection by ECL (Fig. 1A,B) or IR detection (Fig. 1C,D). As seen in Fig. 1, for both VGLUT1 and VGLUT2 the dynamic range of the ECL detection was comprised of between 1 and 5 µg of protein. In contrast, the IR detection was linear between 1 and 20 µg of cortical protein. IR detection linearity was also established within this range of protein concentration for the following proteins: α-tubulin, synaptophysin, PSD95, VIAAT and EAAT2 (Fig. 1E–I). Consequently, the WB studies were conducted with IR detection rather than ECL detection (see examples of blots in Fig. S1A–C).

### Effect of gender, age of death and post-mortem interval on synaptic biomarkers

As shown in Fig. S2, the age of death (AOD) and post-mortem interval (PMI) only minimally impacted VGLUT1, VGLUT2, EAAT2, VIAAT, somatostatin, ChAT, synaptophysin and α-tubulin expression in BA9. In contrast, PSD95 expression levels declined when the PMI increased (Fig. S2, R^2^ = 0.25, *p* = 0.0007). PSD95 variations among the CDR groups should, thus, be interpreted cautiously.

**Figure 2:**
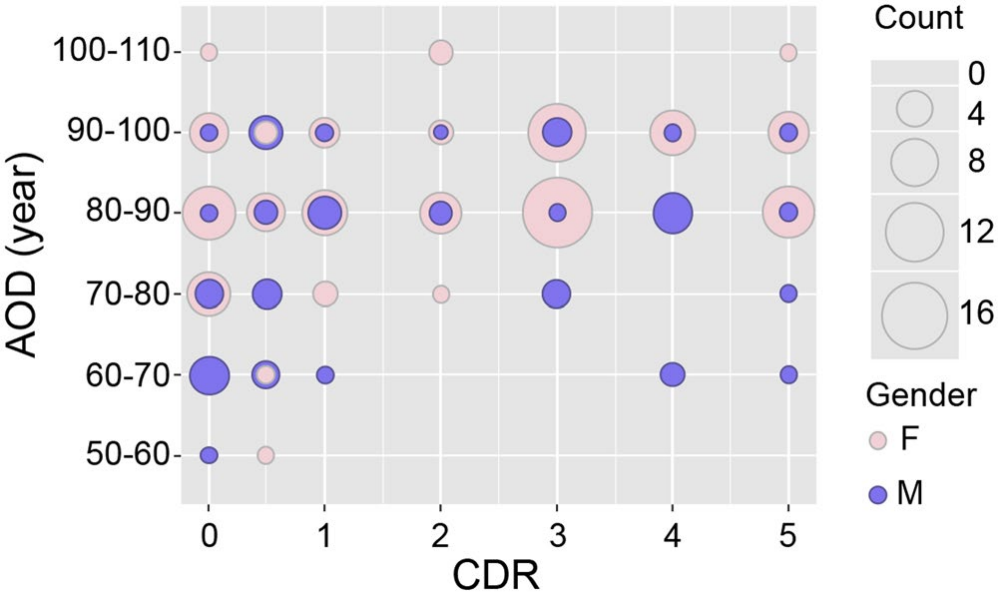
Gender distribution according to the CDR groups (x-axis) and age groups (y-axis). The balloon plots are proportional to the number of individuals in each CDR/age group. Men are shown in semi-transparent violet and women in pink. The number of individuals in each group is proportional to the size of the balloon.

The gender ratio (f/m) varied from 0.8 (CDR0.5) to 4.4 (CDR3) among the groups (Table 1). The distribution of men and women according to their CDR groups and categories of age is shown in Fig. 2. Some CDR/age groups were composed exclusively of men (for example CDR0 (50–60 years old (yo)), CDR1 (70–80 yo), CDR4 (60–80 yo)) or of women (for example CDR0, CDR2 and CDR 5 (>100 yo)). Consequently, the means of the different neuronal markers for men and women could not be compared among the different CDR/age groups. Therefore, marker expression was analysed in all men vs. all women (Fig. S3). A gender effect was never observed, irrespective of the marker studied. The lowest p-values that were found concerned somatostatin expression (t-test *p* = 0.12). Hence no differences were shown between women and men in the overall expression of the different markers studied.

**Table 1:** Characteristics of the subjects from the Mount Sinaï Hospital cohort stratified by CDR scores (mean ± sem).

CDR	N	PMI (min)	AOD (years)	Gender (ratio f/m)
0	38	749.3 ± 11.7	78.4 ± 0.3	27 f/11 m (2.5)
0.5	21	521.9 ± 19.6	81.4 ± 0.5	9 f/12 m (0.8)
1	18	423.7 ± 22.3	85.7 ± 0.5	12 f/6 m (2)
2	14	485.1 ± 25.3	89.5 ± 0.5	11 f/3 m (3.7)
3	38	426.0 ± 8.0	88.2 ± 0.2	31 f/7 m (4.4)
4	21	351.5 ± 15.9	87.2 ± 0.4	13 f/8 m (1.6)
5	21	346.9 ± 14.9	87.8 ± 0.4	17 f/4 m (4.2)

Abbreviations in Table: CDR: clinical dementia rating; N, size of the sample; PMI(min): post-mortem interval in minutes; AOD: age of death; f/m: females/males. One hundred seventy-one brain samples out of the 182 individuals were used and are presented  above.

### Effect of cognitive impairment on glutamatergic markers in BA9

**Figure 3:**
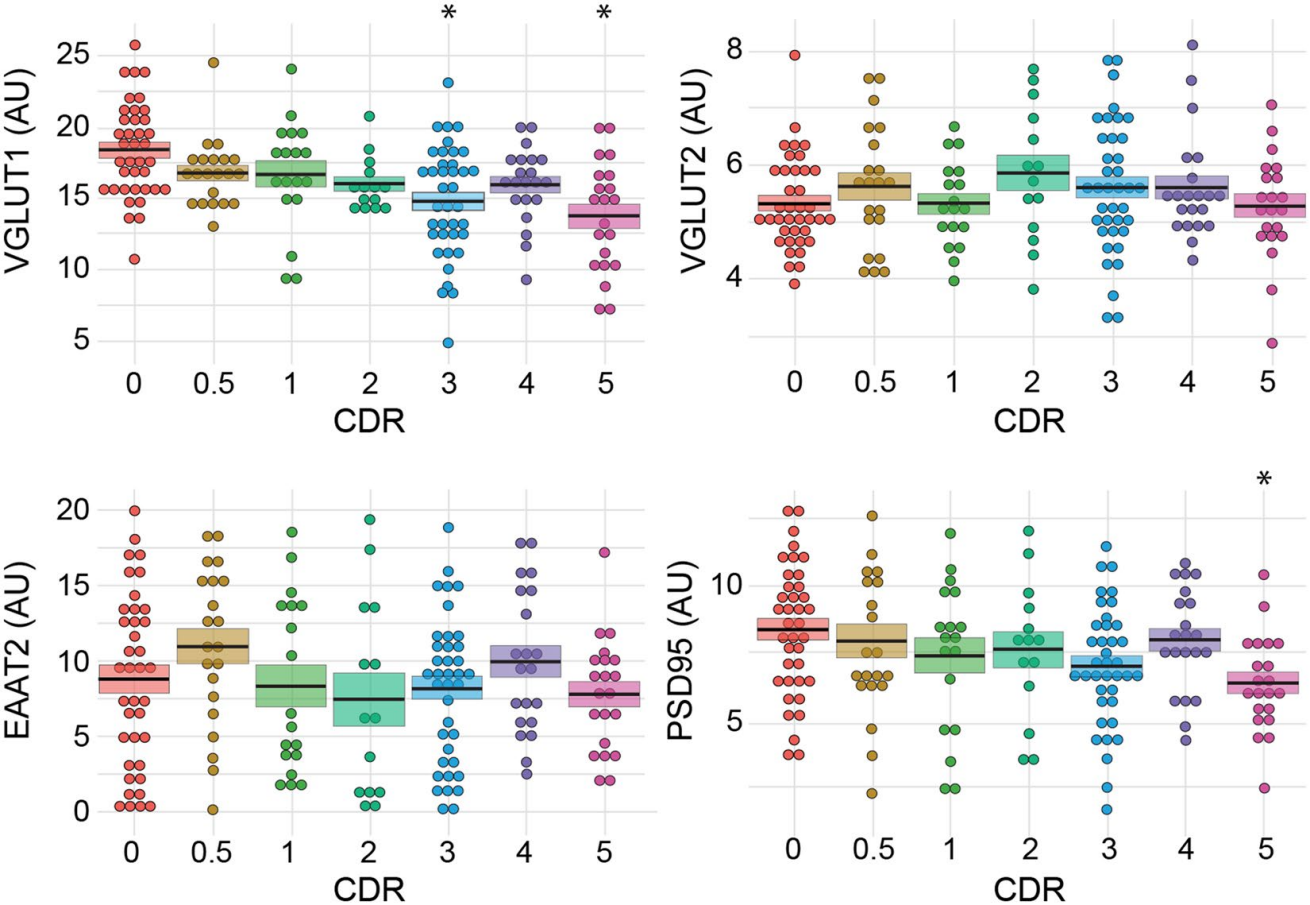
Dot plot representation of the effect of dementia on the expression of glutamatergic markers. VGLUT1, VGLUT2, EAAT2 and PSD95 were measured by western blot coupled to infrared detection and the expression levels (in arbitrary units) were plotted against the CDR scores. Values for each individual are represented by dots. The mean is depicted as the black line in the middle of a box representing the SEM. VGLUT1 expression was significantly reduced at CDR scores of 3 and 5 (*p* < 0.05), whereas VGLUT2 and EAAT2 expression were not modified regardless of the cognitive status of the patient. PSD95 showed a decrease during the last stage only at CDR scores of 5. *p < 0.05.

**Figure 4:**
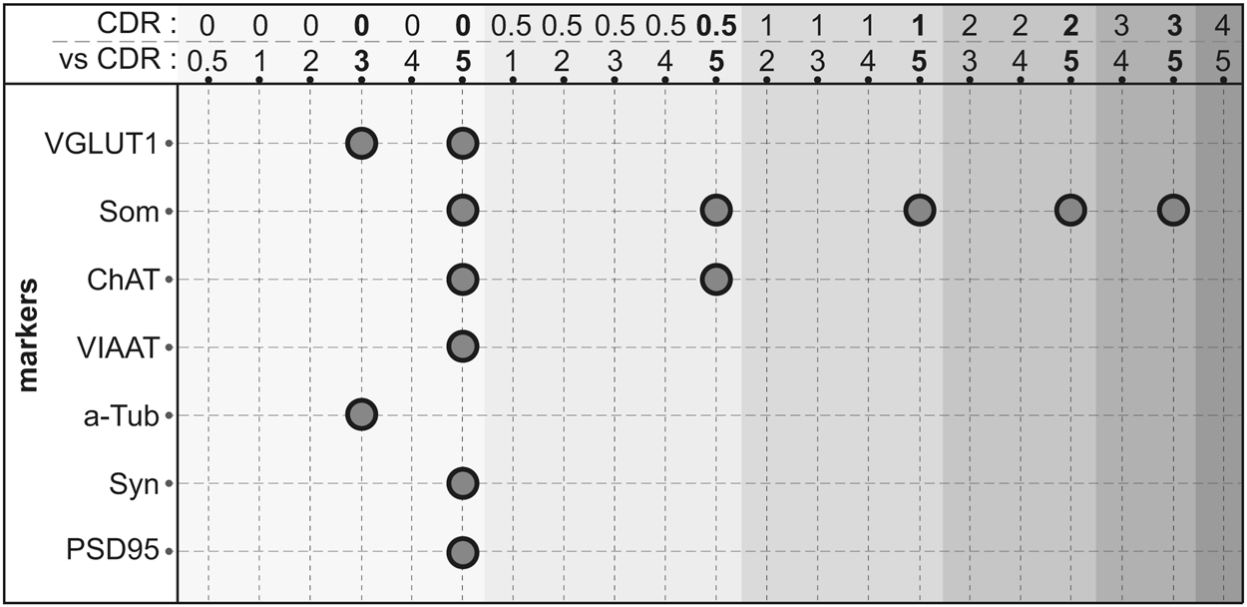
Visual representation of Kruskal-Wallis post hoc analysis output for synaptic markers among CDR scores. The first row represents the value of the CDR couples analysed (i.e., CDR0 vs. CDR0.5 for the first comparison). Significant differences are highlighted for each marker by a dark circle (p < 0.05). Som (somatostatin); α-tub (α-Tubulin); Syn (synaptophysin).

We then compared the amounts of various presynaptic (VGLUT1 and VGLUT2), postsynaptic (PSD95) and glial (EAAT2) markers of glutamatergic transmission in BA9 areas of the subjects stratified according to their CDR scores. As illustrated in Fig. 3, inter-individual differences were important and precluded the use of these markers to diagnose dementia. VGLUT2 and EAAT2 were not affected by dementia (Kruskal-Wallis test, *p* > 0.05; Table 2). In contrast, VGLUT1 and PSD95 were significantly reduced (Kruskal-Wallis test, *p* = 0.0001 and 0.0118, respectively; Table 2). A Kruskal-Wallis post hoc test was performed to identify the CDR groups that were most affected (Fig. 4): VGLUT1 and PSD95 protein expression levels were decreased in individuals with severe dementia (CDR5), but only VGLUT1 expression was significantly decreased in individuals with a CDR score of 3.

**Figure 5:**
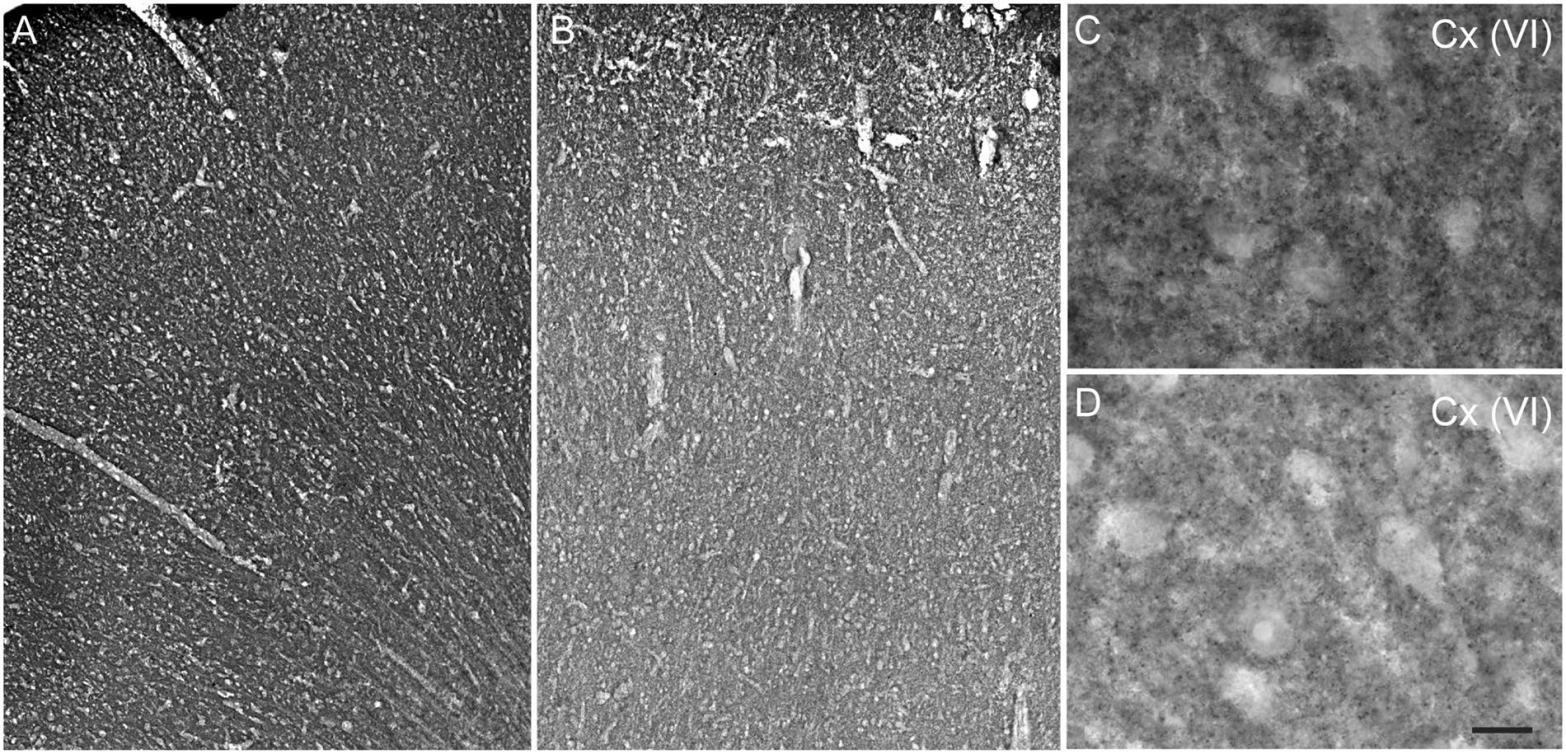
Immunohistochemical detection of VGLUT1 in BA9. Sections from the prefrontal cortex of a control (**A**,**C**) and of a matched patient (**B**,**D**) with severe dementia were immunostained for VGLUT1. Photomicrographs were taken of the entire BA9 layer (**A**,**B**) or at the level of layer VI (Cx(VI) for **C**,**D**). The scale bar in (**D**) represents 150 µm in (**A**,**B**) and 25 µm in (**C**,**D**).

We assessed VGLUT1-positive terminals in BA9 grey matter samples from controls (Fig. 5A,C) and patients (Fig. 5B,D) using immunohistochemistry. As previously reported in the human cerebral and cerebellar cortices^30,51^, the black precipitate formed by VGLUT1 immuno-positive terminals appeared to be very densely packed. Therefore, VGLUT1-positive puncta in all layers of BA9 were hardly quantifiable on the immunostained sections. Interestingly, and as previously reported^30^, the density of VGLUT1-IR seemed to be uniformly decreased in patients (see Fig. 5). We observed no perinuclear accumulation of VGLUT1-positive immuno-material. Therefore, in 2 individuals with cognitive impairment, the density of VGLUT1 per terminal appeared to be decreased in grey matter areas.

### Effect of cognitive impairment on GABAergic markers in BA9

**Figure 6:**
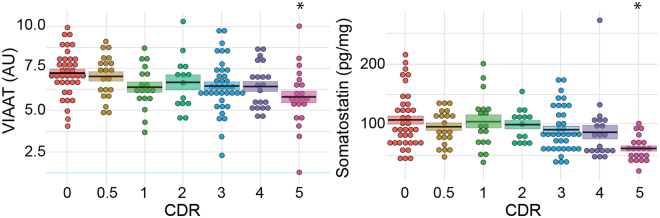
Dot plot representation of the effect of dementia on the expression of GABAergic markers. VIAAT expression levels were determined by western blots, and somatostatin was measured by radioimmunoassay. The results were plotted against the CDR scores. Each individual value is represented by one dot. Bold black lines represent the mean, and colour boxes represent the SEM. VIAAT and somatostatin expression were significantly reduced in subjects with CDR scores of 5 (p < 0.05). *p < 0.05.

We then inspected two GABAergic markers (Fig. 6). VIAAT, the GABA/glycine vesicular transporter, is present in all GABAergic terminals. In contrast, somatostatin is expressed by a restricted population of GABAergic interneurons that densely innervate pyramidal cells in the frontal cortex^52,53^. VIAAT expression levels were found to be similar across the CDR scores of 0–4 (Figs 4 and 6). Only patients with a CDR score of 5 showed a significant decrease in VIAAT protein expression (−21.5%, Kruskal-Wallis test, *p* < 0.05). Somatostatin levels remained stable in samples associated with CDR scores below 5. However, confirming previous findings^37^, when dementia severity increased to a CDR score of 5, somatostatin expression significantly decreased in BA9 (−42.4%, Kruskal-Wallis test, p < 0.05, Figs 4 and 6).

### Effect of cognitive impairment on ChAT in BA9

**Figure 7:**
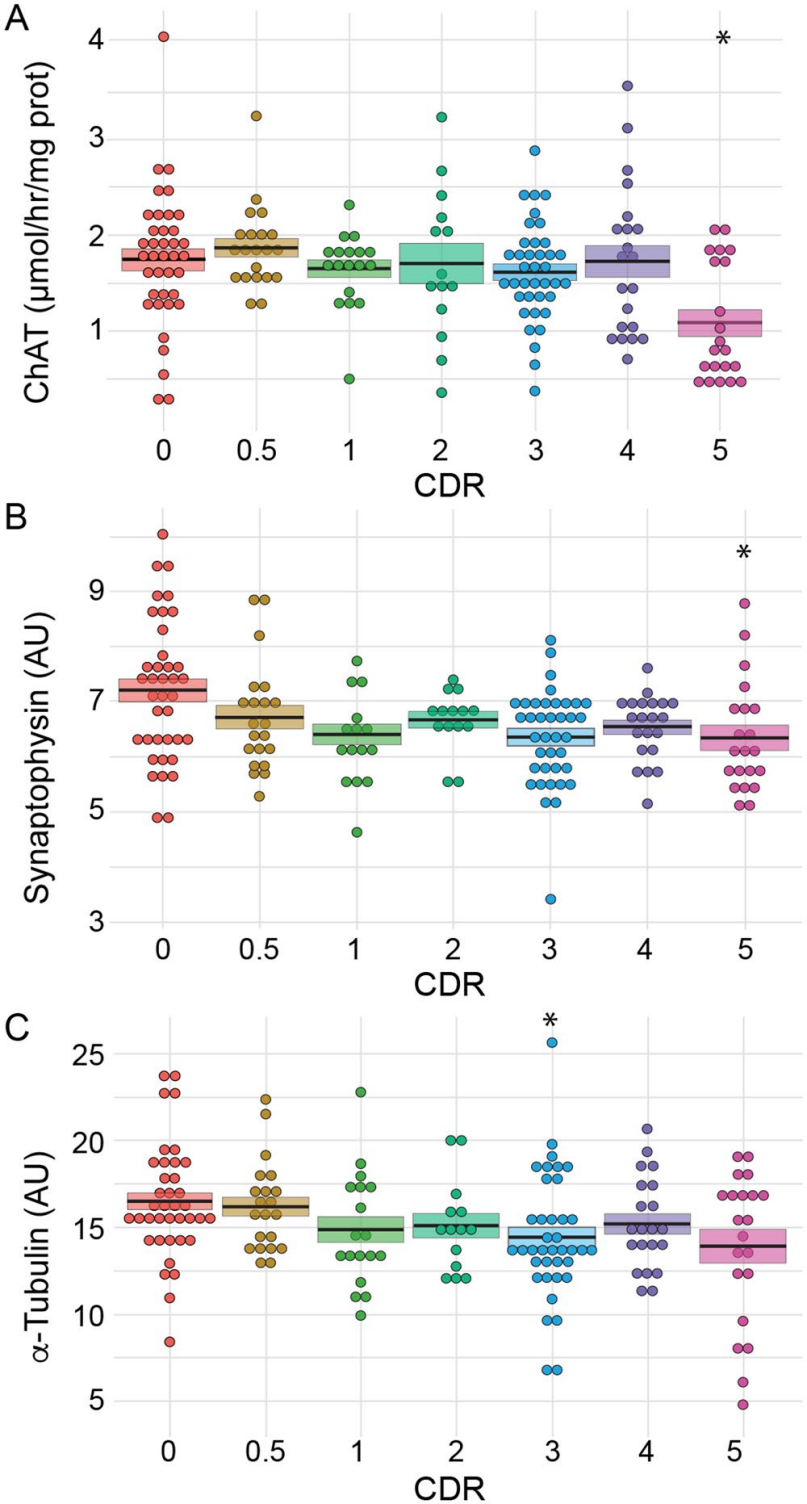
Dot plot representation of the effect of dementia on ChAT, synaptophysin and α-tubulin expression. ChAT activity (**A**) was measured in the BA9 extracts, whereas synaptophysin (**B**) and α-tubulin (**C**) were detected by western blot. Corresponding values were plotted against the CDR scores. Dots represent individual values. The mean is represented by the black line, and the SEM is represented by the coloured box. ChAT and synaptophysin expression were significantly reduced in individuals with CDR scores of 5 (p < 0.05), whereas α-tubulin expression was only modified at CDR scores of 3. *p < 0.05.

A previous post-mortem study established that ChAT activity is strongly decreased in several cortical areas (BA7, 8, 17/18, 20, 21, 22, 24/32, 44) of severely demented subjects^37^. In agreement with this finding, ChAT activity was found here to be significantly decreased (−34.6%, Kruskal-Wallis test, *p* < 0.05, Figs 4 and 7A) in BA9 samples from subjects with severe dementia (CDR5). However, ChAT activity remained unchanged between CDR scores of 0 and 4 (Fig. 7A).

### Effect of cognitive impairment on synaptophysin and α-tubulin in BA9

Synaptic loss is commonly considered as one of the earliest hallmarks of AD. We, therefore, quantified synaptophysin expression by immunoblotting BA9 extracts. Surprisingly, synaptophysin was minimally affected by the progression of dementia (Fig. 7B). Synaptophysin expression levels significantly decreased only in subjects with CDR scores of 5 (−12.1%, Kruskal-Wallis test, *p* < 0.05, Fig. 4).

Alpha-tubulin, the major constituent of microtubules that is expressed by all brain cells, is often used as a loading control in western blots. As shown in Fig. 7C, α-tubulin values decreased moderately during the progression of dementia. A maximal decrease was observed in subjects with a CDR score of 3 (−18.3%, Kruskal-Wallis test, *p* < 0.05, Fig. 4). Therefore, the use of α-tubulin to normalize western blot in AD samples should be considered cautiously.

### Correlation Analysis

**Figure 8:**
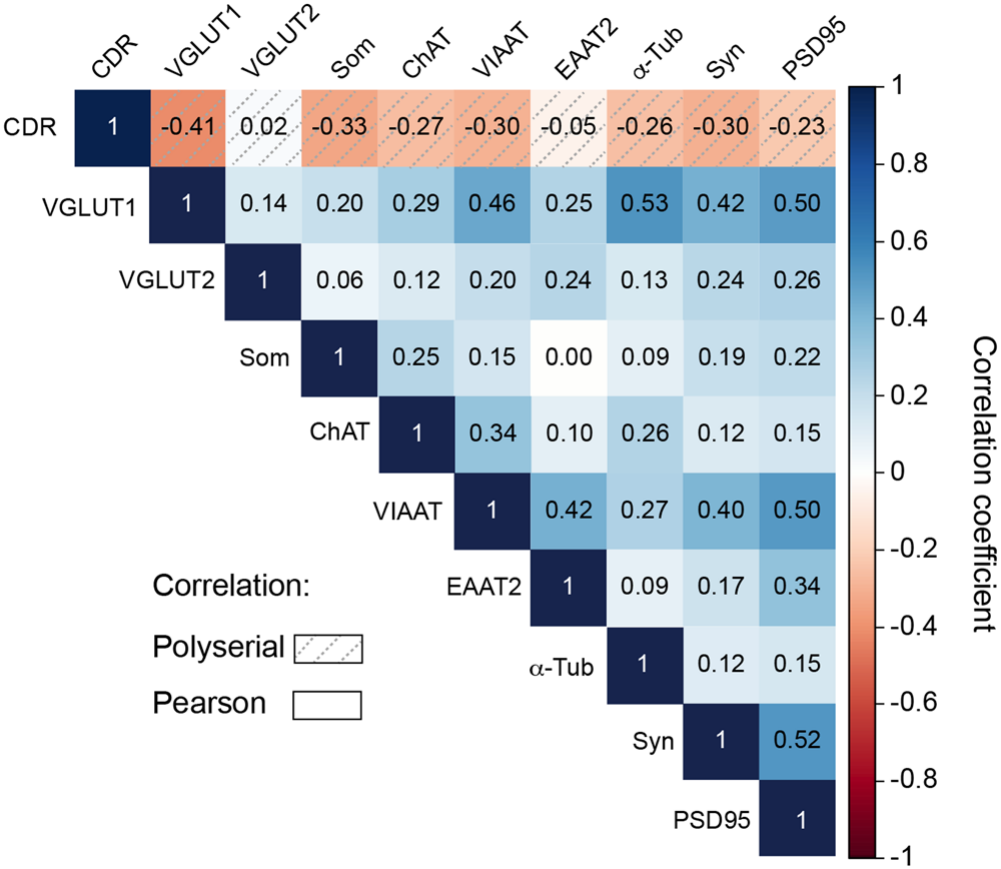
Polyserial and Pearson correlation analyses. Polyserial (ρ) and Pearson (r) correlation coefficients between the CDR scores and VGLUT1, VGLUT2, somatostatin (Som), ChAT, VIAAT, EAAT2, α-tubulin, synaptophysin (syn) and PSD95 were determined. The various correlation coefficients were colour coded from dark blue for a correlation of 1 to a deep red for −1 (and white for 0). Polyserial correlations between the markers and CDR scores are represented by the dashed boxes on the first row. Pearson correlations are presented between the different markers. VGLUT1 shows a negative correlation with the CDR scores, which was higher than that of somatostatin, VIAAT and synaptophysin (r < −0.3). The Pearson correlations highlighted a positive correlation between VGLUT1 vs. α-tubulin, PSD95 and VIAAT (r > 0.46) and between PSD95 vs. VIAAT and synaptophysin (r > 0.5). The *p*-values of the Pearson correlation analyses are presented in Table 2.

The large number of subjects in the present study (n = 171) allowed us to perform a polyserial correlation analysis between all the variables and the CDR scores (Fig. 8, dashed squares on first line). As previously reported with a more limited number of subjects^30^, VGLUT1 had the strongest correlation coefficient with the CDR scores (ρ = −0.41, Fig. 8), followed by somatostatin (ρ = −0.33), VIAAT (ρ = −0.30) and ChAT (ρ = −0.27). The correlation coefficients (ρ) between the CDR scores and VGLUT2 and EAAT2 were very close to 0.

**Table 2:** Kruskal-Wallis test output from the analysis of synaptic markers vs. CDR scores.

Markers	*p*-value
VGLUT1	0.0001
VGLUT2	0.4154
EAAT2	0.1918
PSD95	0.0118
Somatostatin	0.0001
VIAAT	0.0056
ChAT	0.0150
Synaptophysin	0.0313
α-Tubulin	0.0183

In addition, Pearson correlations were performed between all biomarkers (Fig. 8 and Table S2 for p values). The strongest positive correlations were observed between VGLUT1 and α-tubulin (r = 0.53, *p* = 1.55E-13), PSD95 and synaptophysin (r = 0.52, *p* = 4.83E-13), VGLUT1 and PSD95 (r = 0.50, *p* = 3.83E-12), VIAAT and PSD95 (r = 0.50, *p* = 2.68E-12) and VGLUT1 and VIAAT (r = 0.46, *p* = 3.64E-10). These observations suggest a complex, although weak, interplay between neurotransmission markers and dementia.

### Principal Component Analysis

**Figure 9:**
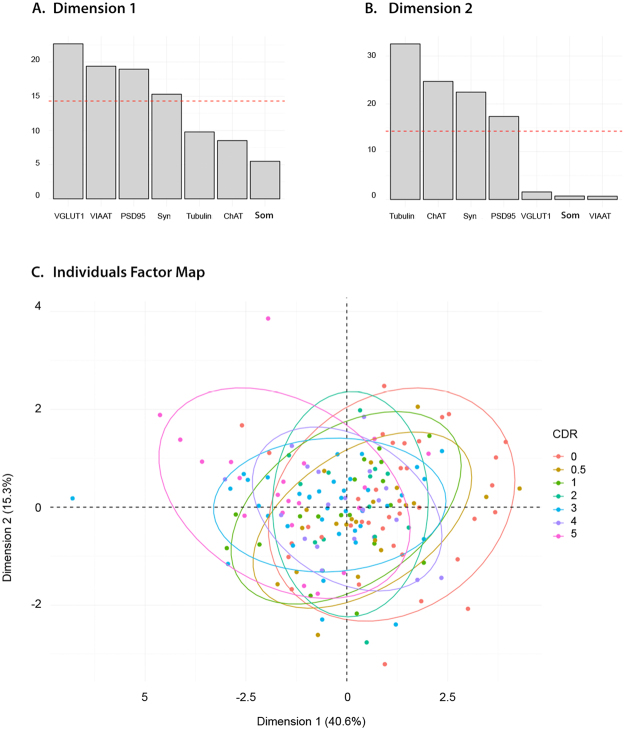
Principal component analysis. The contribution of the variables to the first (**A**) and second (**B**) dimensions. VGLUT1 was the major contributor to the first dimension (23%) and α-tubulin for the second dimension (32%). The individual factor map (**C**) illustrates the principal component analysis. No clear relationship emerged between biological markers represented by the 2 dimensions and the CDR scores.

As described above, the 9 markers assessed in the BA9 samples appeared to be only weakly associated with early stages of cognitive impairment (CDR 0–4). Thus, we explored the possibility of a linear combination of the different markers segregating the different CDR groups. Therefore, we combined 7 markers for which a difference between the groups was detected (VGLUT1, VIAAT, somatostatin, ChAT, α-tubulin, synaptophysin and PSD95 (Fig. 4)) into a Principal Component Analysis (PCA^54^), (Fig. 9) to further explore our dataset. As shown on the scree plot (Fig S4), the first two principal dimensions explained 56.4% of the total variance of these 7 markers. Dimensions 1 and 2 accounted for only 40.6% and 15.8% of the variance, respectively. VGLUT1 (≈23%), followed by VIAAT and PSD95 (both approximately 18%), were the major contributors to the variance in the first dimension (Fig. 9A). This suggested that these 3 markers are somewhat involved in cognitive impairment, albeit weakly. The variance of the second dimension was mainly influenced by α-tubulin (≈32%), ChAT (≈25%), and synaptophysin (≈22%) (Fig. 9B). As shown on the PCA biplot (Fig. 9C), where the samples are projected onto the first two principal components, the seven CDR groups overlapped widely. This absence of clear clusters indicates that the linear combination of the biomarkers used in this study could not be used to define the CDR groups. The score plot in Fig. 9C shows no clear shift in the distribution pattern of the marker expression levels.

## Discussion

Synaptic loss has long been considered among the key hallmarks of AD^8,12,13,55^. The aim of the present study was to investigate the status of glutamatergic, GABAergic and cholinergic synaptic markers in a large cohort (171 subjects) of samples from prefrontal cortex (BA9) extract stratified by the CDR scores of the subjects. BA9 is a major integrative cognitive area of the prefrontal cortex, and previous results have suggested that it is strongly affected in dementia^30,56,57^.

Whether synapses are lost in the cortex during normal ageing has been a matter of controversy^8,58^. The group of control subjects (CDR0) included 38 individuals aged 59–102 yo. As illustrated in Fig. S2, normal ageing had no effect on the expression of VGLUT1, VGLUT2, PSD95, EAAT2, VIAAT, somatostatin, ChAT, synaptophysin and α-tubulin. Interestingly, in our control group, most of the biomarkers were insensitive to a PMI ranging between 240 and 1437 minutes. Only PSD95 values were correlated with PMI (Pearson, R^2^ = 0.2573, p < 0.001). These observations suggest that, while presynaptic proteins are rather stable, dendritic spines are more labile in post-mortem tissues.

The investigated synaptic markers were either negatively correlated to or not affected by dementia. None of their expression levels were significantly increased during the progression of dementia. These data confirm and extend previous findings showing that AD and dementia result in a loss of synaptic proteins (for review see ref.^12^). Synaptic proteins are not all equally affected by AD^12,14^ or AD-like pathology in mouse models^15,27,28^. However, the extent of the decrease in the biomarkers observed here was less pronounced than previously described (see for example^12,30^). In the present study, at the most severe stages of dementia, VGLUT1, VIAAT and synaptophysin were reduced by only 26.4%, 21.5% and 11.9%, respectively. Therefore, synaptophysin, a major and widespread synaptic protein, was only minimally altered in the BA9 samples of the most severely demented patients (CDR5). A severe loss of cholinergic neurons has been documented in the temporal lobes of AD patients^42^. In the prefrontal lobes, Kashani and colleagues (2008) described a 30% decrease in ChAT expression levels in BA9. These findings were confirmed in the present study since we observed a 34.6% decrease in the expression levels of ChAT during the latest stage of dementia (CDR5).

GABAergic interneurons control and synchronize the activity of glutamatergic networks and are instrumental in information processing and memory^59–61^. In the CDR5 group, GABAergic terminals labelled with VIAAT declined in the same proportion as VGLUT1 (≈−20%). Therefore, the glutamate/GABA balance seems to be globally preserved in AD patients. However, the sharpest decline observed in the present study concerned somatostatin (−42.4%), a finding that was in line with previous investigations showing a reduction ranging between 25 and 66%^38,39,62^. VIAAT is present in all GABAergic terminals, whereas somatostatin is only found in a subpopulation of inhibitory interneurons (also known as Martinotti cells^63^). Our results, therefore, suggested that different subpopulations of GABAergic interneurons may display differential sensitivity to AD, and therefore, the glutamate/GABA coupling may well be altered in patients with severe dementia, as was already shown in AD mouse models^48–50^.

Glutamatergic transmission has been the focus of numerous studies in the field of AD^64,65^. Canonical glutamatergic neurons from the human cortex express either VGLUT1 or VGLUT2^51^. In previous studies, cortical VGLUT2 was either severely affected by AD and dementia (−50%^30^) or unaffected^26^. In the present study, we confirmed that cortical VGLUT2 is not affected at any stage of dementia (−0.3%). In the cortex, VGLUT2-immunopostive terminals emerge from layer V pyramidal neurons and from thalamo-cortical axons^51^. These neuronal pathways appeared to be minimally affected by dementia and AD.

EAAT2 is essentially expressed in astrocytes that are spared during normal brain senescence but are associated with degenerating neurons and senile plaques^66^. The status of EAAT2 expression in the AD brain has not been clearly established^67–69^. We found no modification of EAAT2 in the BA9 samples of AD subjects with various levels of dementia. Therefore, the excitotoxicity hypothesis of AD^23–25^, which is related to a decreased clearance and reduced expression of EAAT2, was not supported by the findings of the present study.

VGLUT1, the major subtype of vesicular glutamate transporters, supports 80% of vesicular glutamate uptake in the mouse brain^70,71^. In the human brain, this protein is expressed mostly (but not exclusively) by cortical neurons^51^. VGLUT1 was previously found to be severely decreased in AD and to be highly correlated with cognitive status^26,30^. These results were only partially reproduced here since we only observed a 26.4% reduction in VGLUT1 expression levels. As shown in Fig. 3, there was an important interindividual dispersion of the VGLUT1 expression levels. For instance, in the control group, the VGLUT1 expression levels varied between 10 and 25 AU and between 7 and 20 AU in the most demented subjects (CDR5). This dispersion could either reflect an actual interindividual variability or could be related to pre-mortem conditions. The mRNA integrity number (RIN) and the pH level of the tissue are parameters that could influence the density of membrane proteins. Future studies should investigate the impact of RIN and pH on the stability of VGLUT1, VGLUT2, PSD95, VIAAT or synaptophysin expression. Nonetheless, there was a large overlap between controls and patients that clearly precluded the use of VGLUT1 as a reliable biomarker of dementia.

Interestingly, the decrease in VGLUT1 expression was more sustained than the decrease in synaptophysin expression (−11.9%). This result suggests that VGLUT1 and synaptophysin are only partially co-localized. Alternatively, and more likely, this finding could reflect the fact that pathological ageing differentially regulates these 2 synaptic markers. Immunohistological analysis of the BA9 sections (Fig. 5 and^30^) suggested that in grey matter areas, a decline in VGLUT1 density per terminal occurred rather than a reduction in the number of VGLUT1-immunopositive terminals. These observations suggest that, in AD, synaptic terminals are physically spared but functionally impaired. However, given the dispersion of the VGLUT1 expression levels, these observations should be interpreted with care and deserve further investigation to be confirmed.

PSD95-positive dendritic spines are often apposed to VGLUT1-postive terminals^72^. Interestingly, in the BA9 samples of demented patients (CDR 5), PSD95-IR dendritic spines exhibited the same extent of reduction as the VGLUT1-positive terminals (≈26%). Furthermore, it should be highlighted that these two markers were highly intercorrelated (r = 0.5, *p* = 0 Fig. 8). Therefore, AD seemed to impact VGLUT1-positive terminals and associated dendritic spines similarly.

Serial correlation and PCA confirmed that, among the synaptic variables assessed in this study, VGLUT1 was the best potential biomarker of dementia^30^. However, the magnitude of its decrease was half of what we initially reported in a smaller sample^30^. The way in which a 26% reduction in VGLUT1 expression impacts glutamatergic transmission is not clear. Data from the literature has suggested that vesicular accumulation of glutamate catalysed by VGLUT1 is not proportional to the amount of transporters present in synaptic vesicles^71,73,74^. It has been suggested that a single VGLUT1 protein per vesicle could be sufficient to fill synaptic vesicles and maintain a normal quantal size^71,73,74^. However, using VGLUT1 heterozygous mice, Balschun and colleagues found a reduction in hippocampal LTP accompanied by specific spatial memory deficits^33^. Thus, it is difficult to evaluate how a 26% reduction of VGLUT1 expression could alter glutamatergic transmission in the BA9 areas of demented subjects. Our PCA results supported a minimal effect of such a reduction. However, it was recently suggested that VGLUT1 performs additional synaptic functions of the known vesicular glutamate loading. Indeed, VGLUT1 is involved in the intersynaptic exchange of synaptic vesicles^75^. Whether a limited decrease in VGLUT1 expression impacts synaptic vesicle exchanges and whether synaptic vesicle exchange directly impacts neurotransmission are still open questions.

Overall, the results of the present study suggest that the loss of synaptic proteins in crude BA9 extracts is limited. However, our data should be interpreted with care since this study analysed the total homogenate of the BA9 grey matter. This approach allowed us to quantify various biomarkers in a large number of samples. However, it precluded a more specific layer-by-layer synaptic analysis. Therefore, our findings do not rule out the occurrence of subtle lamina-specific changes in synapses. However, our results suggest that there is not a dramatic change in synaptic markers in cortical grey matter.

In future studies, it will be important to assess whether synaptic markers are more profoundly altered in other brain areas, such as the hippocampus or entorhinal or temporal cortices. Interestingly, our data show that, with a few exceptions, the majority of significant changes were observed only in severe dementia (CDR5). This observation suggests the following: (i) something dramatic occurs at synapses during the transition from CDR 4 to CDR 5, and (ii) most of the synaptic variables examined in this study are not causal in earlier stages of dementia (CDR 0.5–3).

PCA was used to determine the key variables in the present multidimensional dataset. As shown in Figure S4, the first component explained 40.6% of the total variance of the seven biomarkers that varied to some extent among the CDR groups. Along this dimension, VGLUT1, VIAAT and PSD95 seemed to be the most influential. However, as can be visualized on the PCA biplot in which the dataset was projected onto the two first components (Fig. 9C), a linear combination of variables could not explain the CDR score of a patient. Therefore, the various synaptic markers used in this study, whether taken separately or in combination, did not seem to play a pivotal role in the development of dementia.

These observations suggest that AD is a complex disease involving either a large number of limited changes in multiple markers or non-linear interactions among these markers. Our data show a limited effect of AD on markers of terminals, dendritic spines and astrocytes in BA9. Overall, the present investigation only weakly supported the notion that dementia is associated with a sustained age-dependent loss of synaptic markers in BA9.

## Material and Methods

### Demographic

The post-mortem brains from subjects who participated in studies of ageing and early dementia were received over a period of 20 years by the Mount Sinai School of Medicine Department of Psychiatry Brain Bank^3^. All patients involved signed consents. Diagnostic and dementia assessment consent procedures were approved by the institutional review boards of Mount Sinai Medical Center, Jewish Home and Hospital, and the J.J. Peters Veterans Affairs Medical Center. Consents for brain donation were obtained in writing from the legal next of kin of all donors. Studies on post-mortem brain tissue were performed in compliance with and following approval by the local ethical committee (DC-2008–567). Western blot and biochemical detections were performed on post-mortem prefrontal cortex (BA9 area) samples from 171 individuals provided by the Mount Sinai School of Medicine Department of Psychiatry Brain Bank (now the Mount Sinai NIH Brain and Tissue Repository; Table 1)^3^. The cognitive status of each subject was evaluated during the last 6 months of life with the clinical dementia rating (CDR) scale^76^. CDR scores were as follows: 0 = no dementia, 0.5 = questionable, 1 = mild, 2 = moderate, 3 = severe, 4 = profound and 5 = terminal dementia^77^. The samples were stratified by the CDR scores with 14–38 subjects in each category, with mean age of death (AOD) ranging between 78.4 ± 0.3 (for CDR0) and 89.5 ± 0.5 (for CDR2) (Table 1).

Immunohistochemistry experiments (IHC) were conducted on the brain samples from 3 controls and 3 patients provided by the Douglas-Bell Canada Brain Bank (DBCBB; Table 3).

**Table 3:** Characteristics of the subjects from the Douglas-Bell Canada Brain Bank (DBCBB) samples.

Diagnosis	PMI (min)	AOD (years)	Gender
Control	1369.8	89	Male
Control	1620	81	Female
Control	1605	85	Male
AD senile	1545	84	Male
AD senile	1500	83	Female
AD senile	1420.2	88	Male

Abbreviations in Table: PMI(min): post-mortem interval in minutes; AOD: age of death.

### Preparation of brain tissue samples

BA9 grey matter was dissected from flash-frozen coronal sections pulverized at −80°, as described previously^3^. Frozen powdered tissues were homogenized with a Potter-Eveljhem and then sonicated (3 × 10 sec Power 60 Vibra-Cell^TM^ 72446, Sonic USA) in phosphate buffered saline buffer (PBS) containing protease inhibitors (complete, Roche, France). Total BA9 extracts were stored in aliquots at −80 °C until usage; aliquots were unfrozen only once. Protein concentrations were measured with the Bio-Rad protein assay kit (Bio-Rad, France). Protein concentrations in post-mortem extracts from the controls (CDR 0) and the subgroups of different CDR scores were not significantly different (data not shown).

### Western blots

Westerns blots were performed as previously described^30,78^ with the following modifications. Total BA9 extracts were used for the western blot experiments. Equal amounts of protein (5–10 µg per lane) were separated (4 h, 120 volts) by SDS-PAGE (NuPAGE® Novex® Bis-Tris precast gels, Invitrogen, Life Technologies, France) and electrotransferred (40 min at 100 V) in Tris (50 mM) borate (50 mM) onto nitrocellulose membranes (0, 22 µm pore size, Life Technologies). Protein loading was controlled by reversible Ponceau red staining (Sigma-Aldrich, France). Non-specific sites on the nitrocellulose membranes were blocked for 1 h at room temperature with either PBS containing 0.1% Tween 20 and 5% non-fat dry milk (for VGLUT1 and α-tubulin detection) or PBS containing 0.5% Tween 20, 5% non-fat dry milk and 5% bovine serum albumin (for VGLUT2, synaptophysin, PSD95, VIAAT or EAAT2 detection). Nitrocellulose membranes were incubated overnight with primary antisera at 4 °C in PBS containing 0.1% Tween 20 and 1% non-fat dry milk (for VGLUT1 and α-tubulin detection) or in PBS containing Tween 20 (0.5%), 1% non-fat dry milk and 1% bovine serum albumin (for VGLUT2, synaptophysin, PSD95, VIAAT and EAAT2 detection) (Table 4).

**Table 4:** Antisera used for western blots.

Antiserum	Host	Source/reference	Cat #	Dilution
**Primary antisera:**				
VGLUT1	Rabbit	Homemade^30,51^	n.a.	1:10,000
VGLUT2	Rabbit	Homemade^30,51^	n.a.	1:800
VIAAT	Rabbit	Gift from B.Gasnier^86^	n.a.	1:10,000
α-tubulin	Mouse	Sigma Aldrich	T5168	1:500,000
EAAT2	Mouse	Santa Cruz Biotechnology	sc-365634	1:20,000
PSD95	Mouse	Millipore	MAB1596	1:10,000
Synaptophysin	Mouse	Synaptic System	101 111	1:40,000
**Secondary antisera:**				
Horseradish peroxidase	Mouse	Sigma Aldrich	A9044	1:20,000
Horseradish peroxidase	Rabbit	Sigma Aldrich	A6153	1:20,000
IRDye 700DX	Mouse	Rockland	610–130–124	1:5,000
IRDye 800	Rabbit	Rockland	611–131–122	1:5,000

Western blots were visualized with either enhanced chemiluminescence (ECL) or infrared detection (Fig. 1). For ECL, bound primary antibodies were detected with horseradish peroxidase (HRP)-conjugated anti-rabbit or anti-mouse IgG antisera (Sigma, 1:20,000) and visualized by chemiluminescent detection (SuperSignal West Dura, Pierce, France) on films (Biomax MR Film Kodak) (Table 4). For infrared (IR) detection, bound antibodies were detected with secondary anti-mouse or anti-rabbit IgG antiserums conjugated to IRDye 700 (red display, 1:5,000) or IRDye 800 (green display, 1:5,000) (Rockland) with an infrared imager (Odyssey, Li-Cor, France) (Table 4). ECL films were digitized using a Umax PowerLook 1100 scanner (Umax, Willich, Germany). Optical densities of the ECL or infrared images were determined in arbitrary units using the MCID software (Imaging Research, St. Catharines, Ont., Canada). Samples for each biomarker and subject were run in 4–5 replicates and the average of the replicates was calculated. Standard proteins commonly used as loading controls, such as α-tubulin, β-actin, and glyceraldehyde-3-phosphate dehydrogenase (GAPDH) were found to decrease in AD (for α-tubulin see Fig. 6; β-actin and GAPDH not shown). Therefore, samples were not normalized to classic standards but rather to one standard sample of a control subject that was loaded on each SDS-PAGE gel. Examples of representative blots for the entire cohort and for each biomarker are shown in Supplementary Figure S1, with the control sample mentioned above identified by *.

### VGLUT1 immunohistochemistry

Blocks of frozen BA9 cortical tissue from the post-mortem subjects (Douglas-Bell Canada Brain Bank, DBCBB, Table 3) were fixed overnight at 4 °C in 10% neutral buffered formalin. Tissues were then transferred to a sucrose solution (30% in PBS) for 2–3 days. Blocks were snap-frozen in cold isopentane (−30 °C) and cut (50 µm) using a freezing sliding microtome. Free-floating sections were collected in PBS and stored in an anti-freeze solution (10% glycerol, 10% ethylene glycol in 0.4 × PBS) at −20 °C until used. Sections were incubated with anti-VGLUT1 antiserum (1:500^51,79^) and then processed with biotinylated goat anti-rabbit secondary IgG (1:1000, Vector Laboratories, Burlingame, CA) and streptavidin coupled to horseradish peroxidase (Jackson Immunoresearch, Burlington Ontario, Canada). Immunohistochemical staining was performed as previously described^30^. Images were obtained with a Zeiss Axioscop microscope (Carl Zeiss, Oberkochen, Germany).

### Choline acetyltransferase activity

The choline acetyltransferase (ChAT) activity was measured in the prefrontal cortex BA9 extracts^80^ as previously described^30^.

### Somatostatin radioimmunoassay

Somatostatin-like immunoreactive material was measured by radioimmunoassay as previously described^39,81^.

### Statistical analyses

Statistical analyses and data characterization were conducted in R^82^ version 3.2.2 (2015–08–14)/Platform: x86_64-apple-darwin13.4.0 (64-bit)/Running under: OS X 10.9.5, Mavericks. Kruskall-Wallis post hoc tests were performed using pgirmess R package (version 1.6.5). Figures were generated using the ggplot2 (ref.^83^; version 2.1.0) and factoExtra (ref.^84^; version 1.0.3) R packages for visualization of principal component analysis (PCA). Polyserial combination was performed using the polycor R package^85^.

### Data availability statement

The datasets generated and analysed during the current study are available from the corresponding authors upon reasonable request.

## Electronic supplementary material

Supplementary Materials
