# Supplementary material for: Moderate decline in select synaptic markers in the prefrontal cortex (BA9) of patients with Alzheimer’s disease at various cognitive stages
Source: Sci Rep. 2018 Jan 17;8:938. doi: 10.1038/s41598-018-19154-y (PMC5772053; doi:10.1038/s41598-018-19154-y)
Supplement: Supplementary file 1 — Supplementary Materials [file 41598_2018_19154_MOESM1_ESM.doc]

**SUPPLEMENTARY INFORMATION**

**Moderate decline of selective synaptic markers in the prefrontal cortex (BA9) of patients with Alzheimer disease at various cognitive stages.**

Odile Poirel, Sébastien Mella, Catherine Videau, Lauriane Ramet, Maria Antonietta Davoli, Etienne Herzog, Pavel Katsel, Naguib Mechawar, Vahram Haroutunian, Jacques Epelbaum, Stéphanie Daumas and Salah El Mestikawy

**Figure S1.** Representative western blots for VGLUT1 and α-tubulin (S1-A), VGLUT2, PSD95 and synaptophysin (S1-B), VIAAT and EAAT2 (S1-C).

The aim of the present study was to evaluate the status of biomarkers involved in neurotransmission in the neocortical Brodmann area 9 (BA9) from 171 subjects with different levels of cognitive impairment. VGLUT1&2, EAAT2, PSD95, VIAAT, synaptophysin and α-tubulin were evaluated by western bloting. Infrared WB detection allows detection in 2 different channels. Therefore, western blots of the 7 biomarkers were organized according their molecular weight (MW) and/or to the origin of their primary antiserum (made in rabbit or mouse as depicted in Table 4) as follows: 1) VGLUT1 and α-tubulin (Suppl Fig. S1-A), 2) VGLUT2, PSD95, synaptophysin (Suppl Fig. S1-B) and 3) VIAAT and EAAT2 (Suppl Fig. S1-C). Supplemental Figure 1 (A-C) shows representative experiments for each of the 171 samples and the 7 biomarkers. One sample from a control subject (red asterisk, *****) was used as an internal standard. MW markers (**MW**) were loaded at different positions and in a different number of lanes to identify the blots.

**Figure S1-A**. Western Blot detection of VGLUT1 and α-tubulin


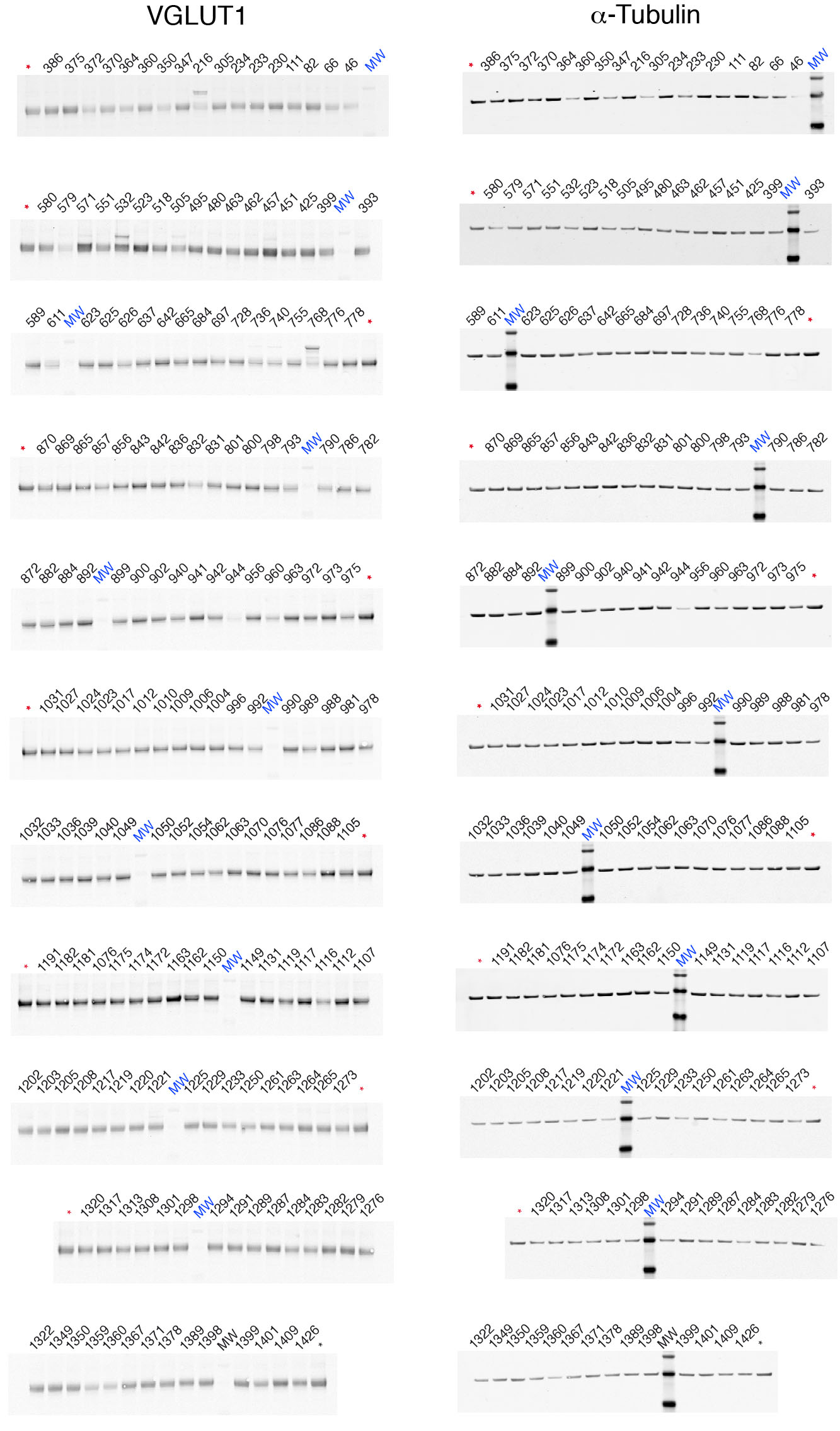


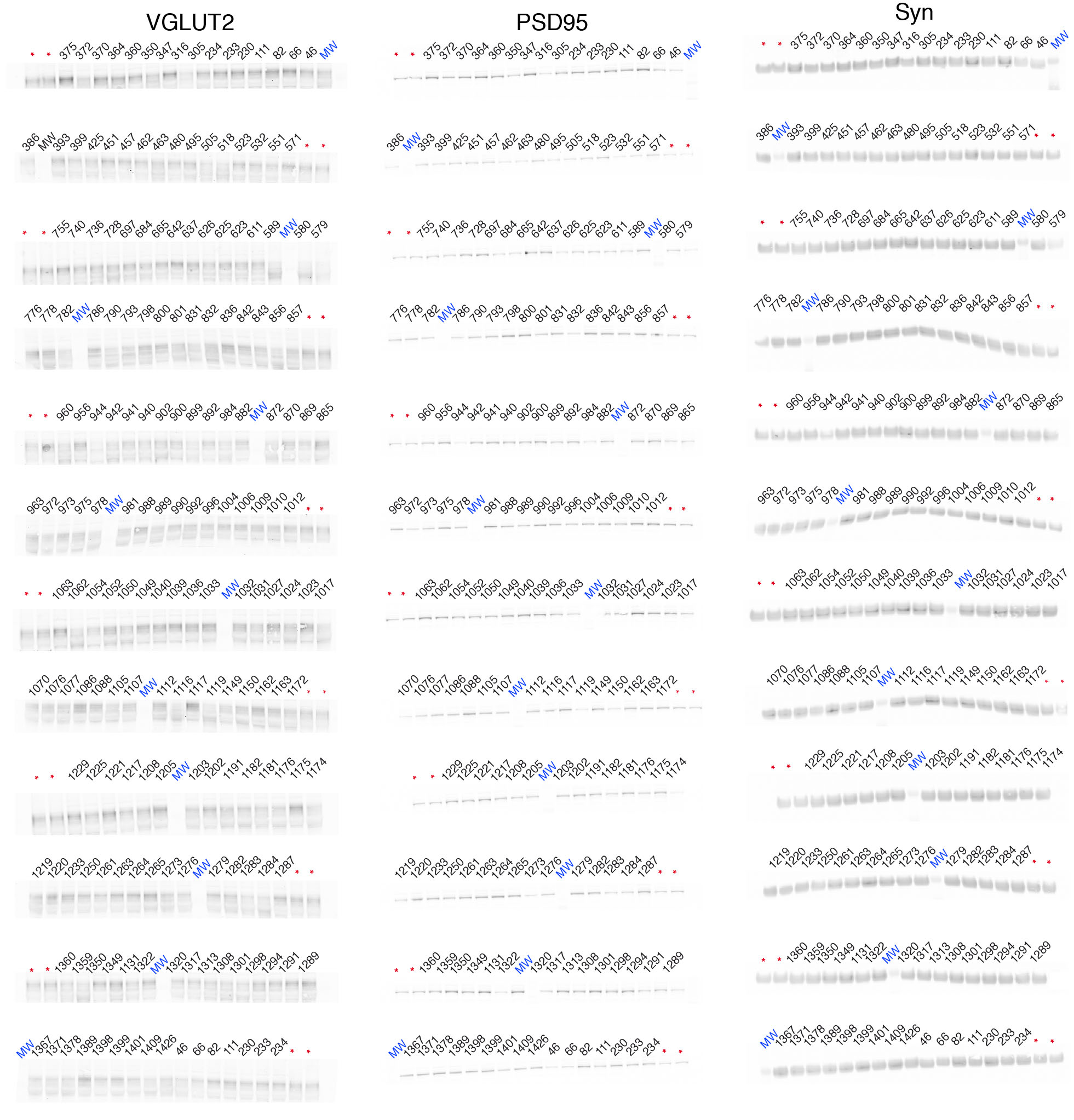
**Figure S1-B.** Western Blot detection of VGLUT2, PSD95 and synaptophysin.


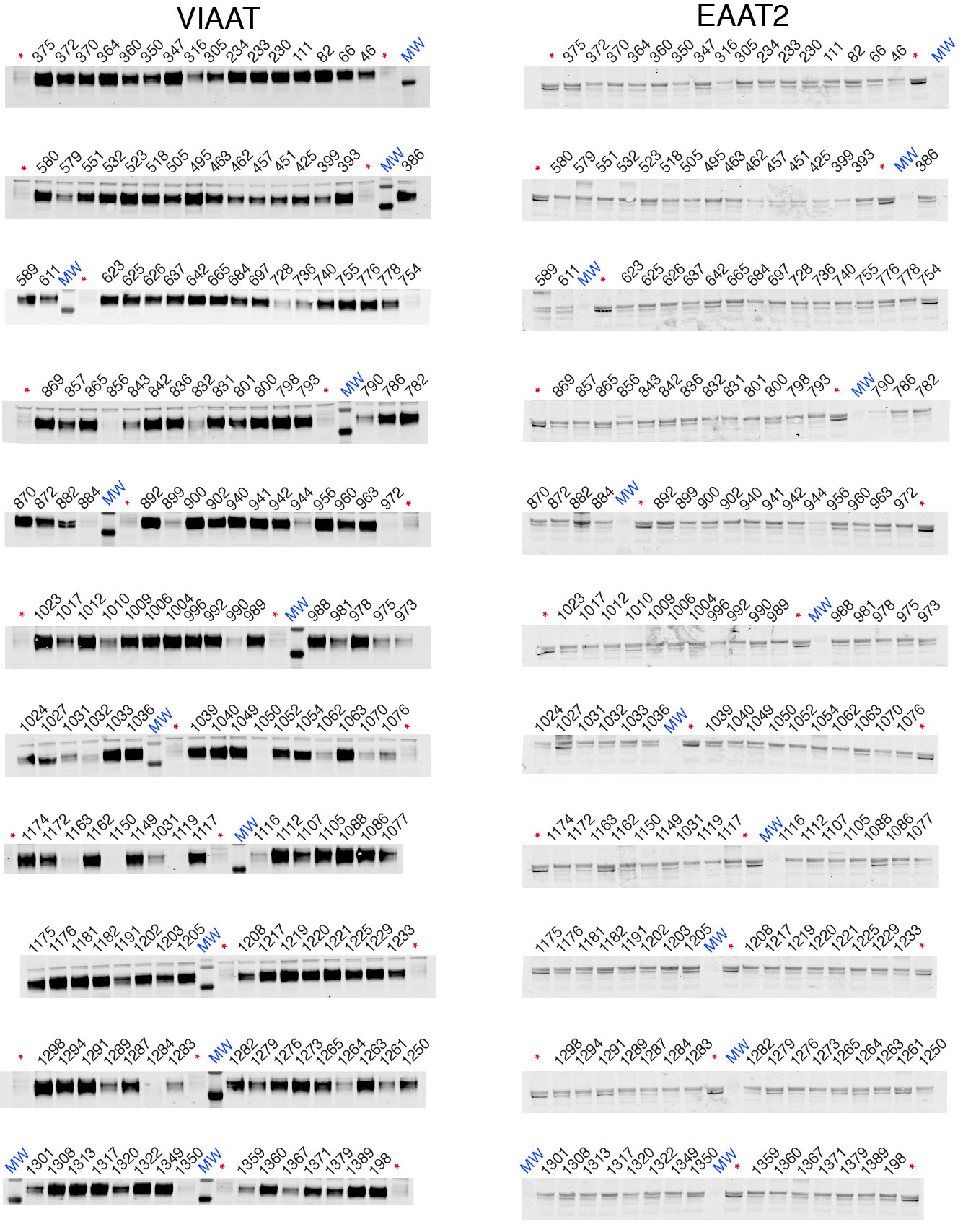
**Figure S1-C.** Western Blot detection of VIAAT and EAAT2.

**Figure S2.** Effect of Age of Death (AOD) and Post Mortem Interval (PMI) on various synaptic markers in the BA9 area of control subjects.

Controls used in this study (subject with CDR 0) consisted of 38 individuals, 27 males and 11 females with age of death (AOD) ranging between 59 and 102 years old (*Table 1*). Their postmortem intervals (PMI) ranged between 140 and 1437 minutes. As shown in Figure S2, AOD or PMI were not correlated with VGLUT1, VGLUT2, EAAT2, VIAAT, somatostatin, ChAT, synaptophysin or α-tubulin expression. Amounts of PSD95 (the postsynaptic marker of glutamatergic synapses) were not affected by aging. In contrast, PSD95 expression significantly declined with increasing PMI (R^2^=0.25, *p* = 0.0007). Therefore, PSD95 variations among CDR groups should be interpreted cautiously. Furthermore, Kruskal-Wallis and pairwise Wilcoxon tests for all the markers in CDR0 group showed no differences in the quantitative variable values whatever the AOD.


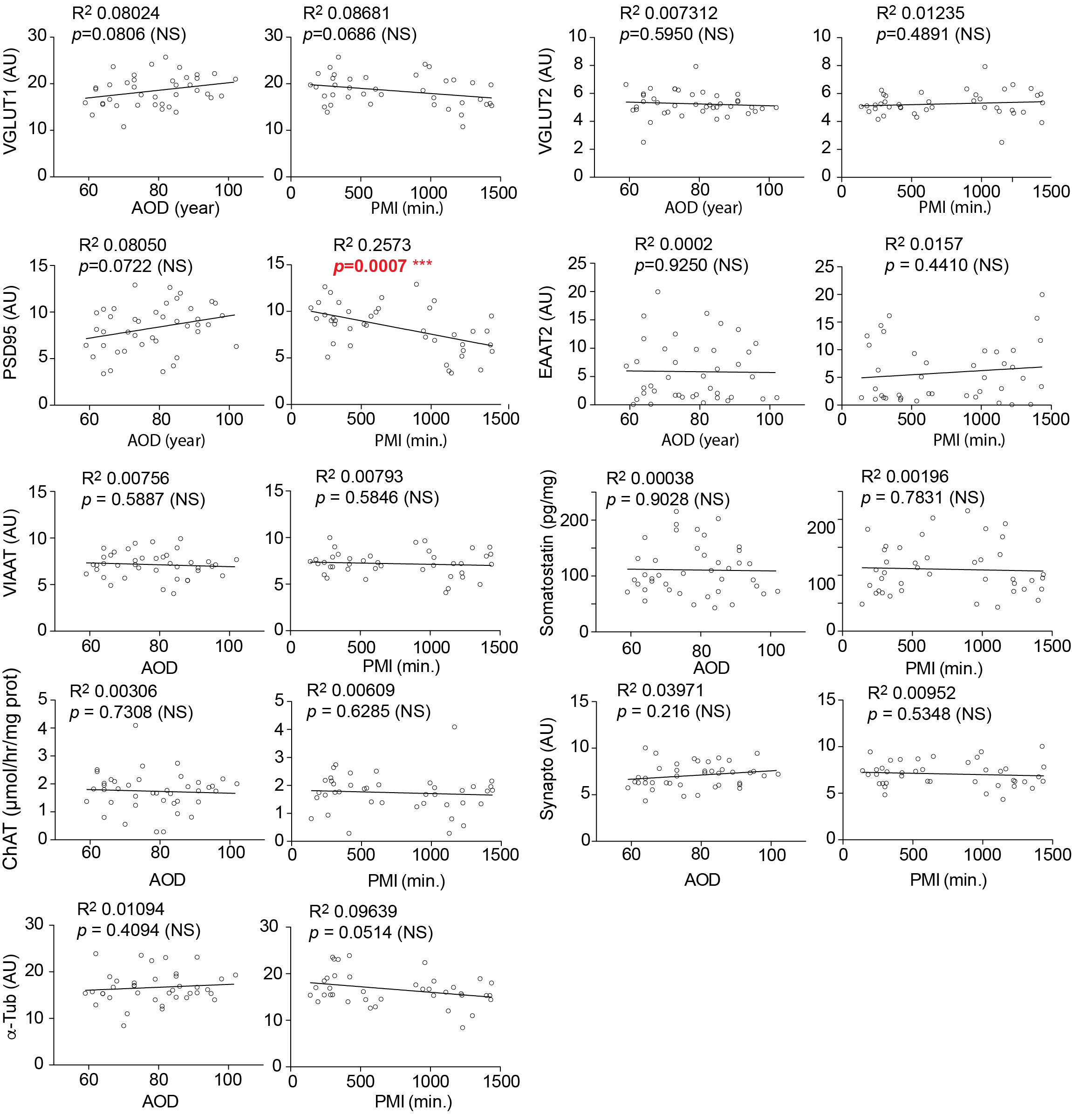
**Legend Figure S2.** Effect of AOD and PMI on various biomarkers in control subjects. The amount of VGLUT1, VGLUT2, PSD95, EAAT2, VIAAT, Synaptophysin and α-tubulin in BA9 area of control subjects were estimated by western blot. Somatostatin was estimated by radiomimmunoassay. ChAT enzymatic activity was measured in BA9 extracts. All values were plotted against age of death (AOD) or postmortem intervals (PMI). PSD95 values only were significantly correlated with PMI. *** *p* = 0.0007.

**Figure S3.** Boxplots showing the distribution for the different neuronal markers in the male and female populations.


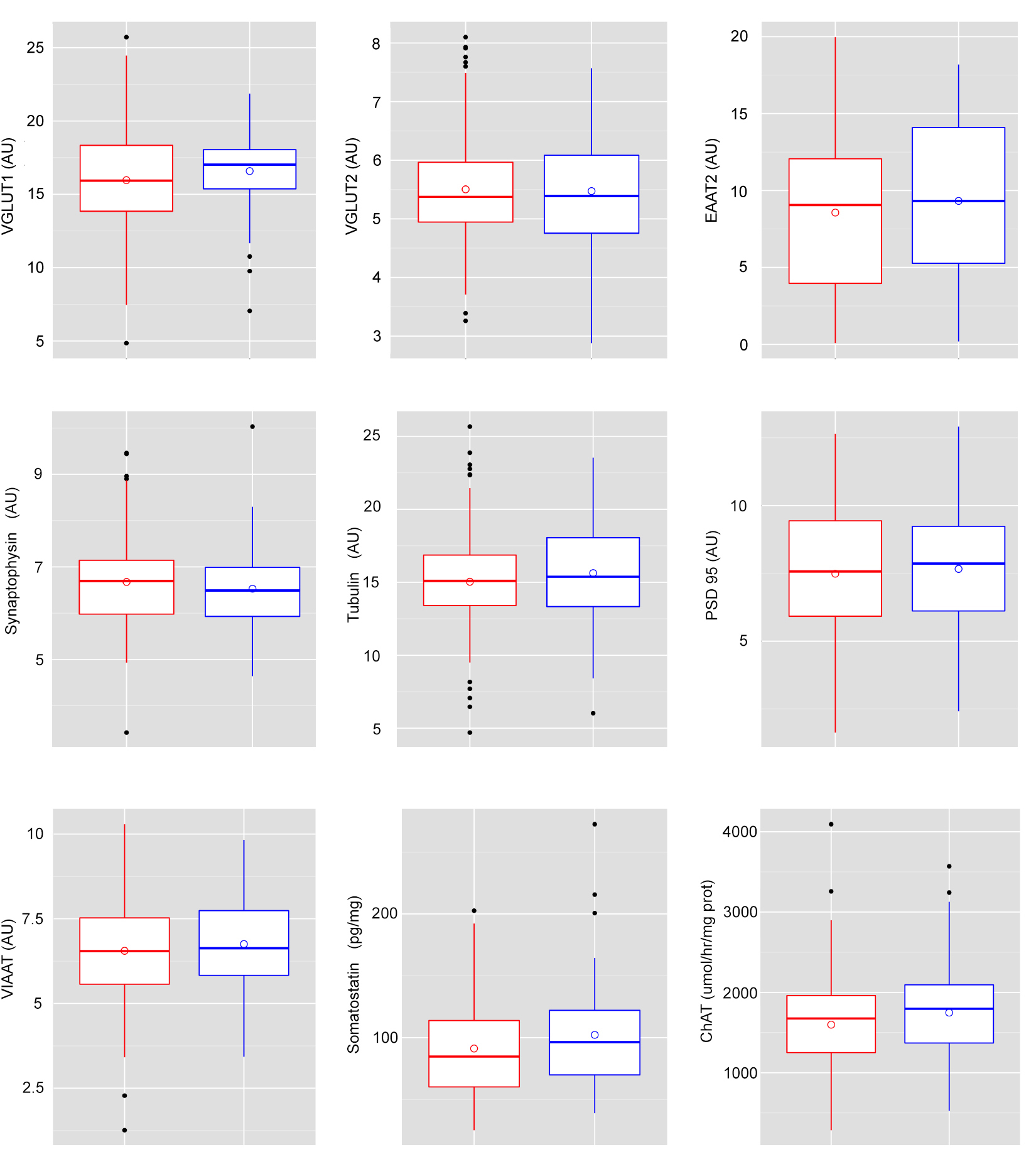


**Legend** **Figure S3.** Red boxplot represents men and blue represents women. Boxes represent the inter-quartile range (IQR). The line inside the box represents the median and the open circle the mean. Outliers (value > 1.5 IQR) are indicated by black dots. All values were plotted depending on the sex of the subjects (all CDRs). There are no differences between women and men in the overall expression of the different markers studied (Pairwise Wilcoxon test output are presented in Table S1).

**Figure S4**. Principal component analysis Scree plot.

Bars represent the proportion of total variance explained by each dimension. Up to 56.4% of the total variance of the seven markers is explained by the first two dimensions (40.6 and 15.8% respectively).


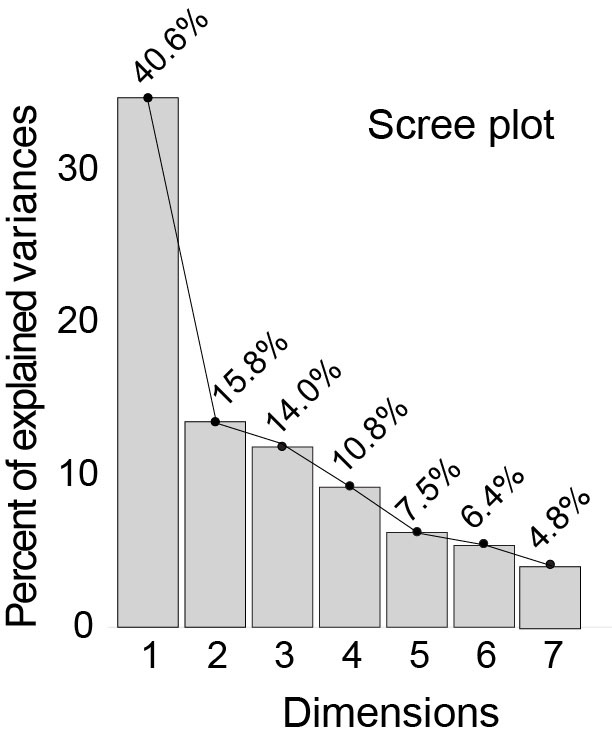


**Table S1 corresponding to Figure S3.** Pairwise Wilcoxon analysis.

| Pairwise Wilcoxon tests for men vs women | |
| --- | --- |
| **Markers** | ***P* Values** |
| VGLUT1 | 0.6570 |
| VGLUT2 | 0.4846 |
| Somatostatin | 0.9103 |
| ChAT | 0.2784 |
| VIAAT | 0.0660 |
| EAAT2 | 0.3395 |
| α-Tubulin | 0.1816 |

**Table S2.** P-values of Pearson correlation coefficients between synaptic markers VGLUT1, VGLUT2, Somatostatin (Som), ChAT, VIAAT, EAAT2, α-tubulin, Synaptophysin (Syn) and PSD95 as presented on Figure 7. Bold number indicate significance (p<0.05)

|  | VGLUT2 | Som | ChAT | VIAAT | EAAT2 | α−Tubulin | Syn | PSD95 |
| --- | --- | --- | --- | --- | --- | --- | --- | --- |
| VGLUT1 | 7,05E-02 | **1,05E-02** | **1,42E-04** | **3,64E-10** | **8,59E-04** | **1,55E-13** | **8,65E-09** | **3,83E-12** |
| VGLUT2 |  | 4,10E-01 | 1,13E-01 | **7,23E-03** | **1,32E-03** | 8,54E-02 | **1,82E-03** | **5,16E-04** |
| Som |  |  | **9,82E-04** | **4,86E-02** | 9,48E-01 | 2,18E-01 | **1,26E-02** | **4,17E-03** |
| ChAT |  |  |  | **6,75E-06** | 2,00E-01 | **7,35E-04** | 1,08E-01 | **4,80E-02** |
| VIAAT |  |  |  |  | **9,94E-09** | **3,56E-04** | **4,66E-08** | **2,68E-12** |
| EAAT2 |  |  |  |  |  | 2,66E-01 | **2,58E-02** | **5,09E-06** |
| α−Tubulin |  |  |  |  |  |  | 1,28E-01 | 5,30E-02 |
| Synaptophysin |  |  |  |  |  |  |  | **4,83E-13** |

**Table S3.** Synaptic markers values according to Neuropathological status

| **Diagnosis (n)** | **VGLUT1** | **VGLUT2** | **PSD95** | **EAAT2** | **VIAAT** | **Somatostatin** | **ChAT** | **Synaptophysin** | **a-Tubulin** |
| --- | --- | --- | --- | --- | --- | --- | --- | --- | --- |
| Normal (63) | 8.8±0.2 | 5.3 ± 0.1 | 7.8 ± 0.3 | 125±11 | 7.0±0.2 | 1.01±0.04 | 1878±93 | 6.8±0.1 | 7.8 ± 0.2 |
| Possible (24) | 8.3±0.3 | 5.7 ± 0.2 | 8.4 ± 0.5 | 133±15 | 7.3±0.2 | 1.15±0.09 | 1679±58 | 6.7±0.2 | 8.2 ± 0.4 |
| Probable (31) | 7.7±0.3 | 5.7 ± 0.2 | 7.0 ± 0.4 | 138±13 | 6.6±0.2 | 1.01±0.05 | 1699±97 | 6.5±0.2 | 7.7 ± 0.2 |
| Definite AD (64) | 7.5±0.3 * | 5.4 ± 0.1 | 7.1 ± 0.3 | 119±10 | 6.0±0.2* | 0.78±0.05* | 1378±75* | 6.4±0.1 | 7.1 ± 0.3 |

* vs NP-1 ANOVA adjusted on AOD and PMI

| **Diagnosis (n)** | **AOD** | **PMI** |
| --- | --- | --- |
| Normal (63) | 78.1±1.4 | 655±57 |
| Possible (24) | 87.8±1.8* | 412±64* |
| Probable (31) | 89.1±1.0* | 423±61* |
| Definite AD (64) | 86.7±1.2* | 399±41* |

Mean ± sem, NS, not significant. * *p* < 0.05

**Table S4.** Overview of all biomarkers quantification in each CDR group.

| **CDR**  **(n)** | **VGLUT1** |  | **VGLUT2** |  | **PSD95** |  | **EAAT2** |  | **VIAAT** |  | **Som** |  | **ChAT** |  | **Syn** |  | **α-Tub** | |
| --- | --- | --- | --- | --- | --- | --- | --- | --- | --- | --- | --- | --- | --- | --- | --- | --- | --- | --- |
|  | Mean±sem  *p* | (%) | Mean±sem  *p* | (%) | Mean±sem  *p* | (%) | Mean±sem  *p* | (%) | Mean±sem  *p* | (%) | Mean±sem  *p* | (%) | Mean±sem  *p* | (%) | Mean±sem  *p* | (%) | Mean±sem  *p* | (%) |
| **0**  **(41)** | 18.5 ±0.5  n.a. | (100) | 5.3±0.1  n.a. | (100) | 8.3±0.4  n.a. | (100) | 8,4±0,9  n.a. | (100) | 7.2±0.2  n.a. | (100) | 110.9±7.1  n.a. | (100) | 1739.5±107.6  n.a. | (100) | 7.1±0.2  n.a. | (100) | 16.6±0.5  n.a. | 100 |
| **0.5**  **(22)** | 17.0±0.5  0.0491 | (91.9) | 5.6±0.2  NS | (106.8) | 8.1±0.6  NS | (97.6) | 11.0±1.1  NS | (120.3) | 7.0±0.3  NS | (97.5) | 95.8±6.0  NS | (86.5) | 1886.5±90.6  NS | (108,4) | 6.8±0.2  NS | (95,7) | 16.4±0.6  NS | 98,8 |
| **1**  **(19)** | 16.6±0.8  NS | (90.0) | 5.3±0.2  NS | (101.3) | 7.3±0.6  NS | (87.9) | 7.9±1.3  NS | (94.8) | 6.4±0.3  0,0263 | (88.3) | 105.6±10.4  NS | (95.3) | 1656.7±92.3  NS | (96) | 6.3±0.2  0,0307 | (89,4) | 14.8±0.7  0,0416 | 89,1 |
| **2**  **(14)** | 16.0±0.5  0,0071 | (86.5) | 5.9±0.3  NS | (111.4) | 7.6±0.7  NS | (91.8) | 7.5±1.7  NS | (91.5) | 6.6±0.4  NS | (92.4) | 100.9±7.1  NS | (91.0) | 1709.5±210.7  NS | (99) | 6.7±0.1  NS | (94,1) | 15.1±0.7  NS | 90,6 |
| **3**  **(40)** | 15.1±0.6  0,0003 | (81.5) | 5.6±0.2  NS | (106.3) | 6.9±0.3  0,0180 | (83.8) | 7.9±10.7  NS | (90.3) | 6.5±0.2  0,0132 | (89.8) | 93.2±5.9  NS | (84.1) | 1641.6±82  NS | (95.1) | 6.3±0.1  0,0117 | (89,8) | 14.4±0.6  0,0023 | 86,7 |
| **4**  **(23)** | 15.5±0.7  0,0046 | (84.0) | 5.7±0.2  NS | (108.2) | 8.0±0.4  NS | (96.3) | 10.1±1.0  NS | (121.4) | 6.4±0.3  0,0283 | (89.1) | 76.6±5.8  0,0033 | (69.1) | 1690.9±159.7  NS | (98) | 6.6±0.2  NS | (93,7) | 14.6±0.7  0,0299 | 88,0 |
| **5**  **(23)** | 13.6±0.8  < 0.0001 | (73.6) | 5.2±0.2  NS | (99.7) | 6.1±0.4  0,0008 | (73,4) | 7,9±0.8  NS | (96.0) | 5.6±0.4  0,0003 | (78.5) | 63.8±4.6  < 0.0001 | (57.6) | 1130.1±132.3  0,0026 | (65.4) | 6.2±0.2  0,0061 | (88,1) | 13.6±0.9  0,0220 | 81,7 |

Abbreviations: Som, somatostatin; Syn, synaptophysin; α-Tub, α-tubulin.

Mean ± sem, NS, not significant. * *p* < 0.05 ; ** *p* < 0.01 ; *** *p* < 0.001 ; *****p* < 0.0001 Mann-Whitney vs CDR0
